# Early administration of levosimendan is associated with improved kidney function after cardiac surgery – a retrospective analysis

**DOI:** 10.1186/s13019-014-0167-8

**Published:** 2014-11-18

**Authors:** Felix Balzer, Sascha Treskatsch, Claudia Spies, Michael Sander, Mark Kastrup, Herko Grubitzsch, Klaus-Dieter Wernecke, Jan P Braun

**Affiliations:** Department of Anesthesiology and Intensive Care Medicine, Campus Charité Mitte and Campus Virchow-Klinikum, Charité - Universitätsmedizin Berlin, Charitéplatz 1, Berlin, 10117 Germany; Department of Cardiovascular Surgery, Campus Charité Mitte, Charité – Universitätsmedizin Berlin, Berlin, Germany; SOSTANA GmbH, Berlin, Germany; Department of Anesthesiology, Intensive Care Medicine and Pain Therapy, Klinikum Hildesheim GmbH, Hildesheim, Germany

**Keywords:** Levosimendan, Kidney function, Renal effects, Cardiac surgery, Left ventricular ejection fraction

## Abstract

**Background:**

Several animal studies suggest beneficial effects on kidney function upon administration of levosimendan. As recent data from clinical studies are heterogeneous, we sought to investigate whether levosimendan is associated with improved postoperative kidney function in cardiac surgery patients with respect to timing of its administration.

**Methods:**

Retrospective, single centre, observational analysis at a university hospital in Berlin, Germany. All adult patients without preoperative renal dysfunction that underwent coronary artery bypass grafting and/or valve reconstruction/replacement between 01/01/2007 and 31/12/2011 were considered for analyses.

**Results:**

Out of 1.095 included patients, 46 patients were treated with levosimendan due to a severely reduced left ventricular systolic function preoperatively (LVEF < 35%) and/or clinical signs of a low cardiac output syndrome. Sixty-one percent received the drug whilst in the OR, 39% after postoperative intensive care unit admission. When levosimendan was given immediately after anaesthesia induction, creatinine plasma levels (p = 0.009 for nonparametric analysis of longitudinal data in a two-factorial design) and incidence of postoperative renal dysfunction (67.9% vs. 94.4%; p = 0.033) were significantly reduced in contrast to a later start of treatment. In addition, duration of renal replacement therapy was significantly shorter (79 [35;332] vs. 272 [132;703] minutes; p = 0.046) in that group.

**Conclusions:**

Postoperative kidney dysfunction is a common condition in patients under going cardiac surgery. Patients with severely reduced left ventricular function and/or clinical signs of a low cardiac output syndrome who preoperatively presented with a normal kidney function may benefit from an early start of levosimendan administration, i.e. immediately after anaesthesia.

**Trial registration:**

Clinicaltrials.gov-ID: NCT01918618.

**Electronic supplementary material:**

The online version of this article (doi:10.1186/s13019-014-0167-8) contains supplementary material, which is available to authorized users.

## Background

Cardiac surgery is associated with a high risk for peri- and postoperative complications [1]. In consequence, protecting organ function in the operative setting plays an important role for improving patient outcome. Postoperative renal dysfunction is associated with increased mortality of 60-90%, and especially in patients undergoing cardiac surgery, prolonged stays on the intensive care unit (ICU) and in the hospital have been reported [2].

Levosimendan is a long acting calcium sensitizer derived from pyridazinone-dinitrile [3]. It enhances contractility without increasing oxygen consumption by calcium concentration-dependent conformational changes in troponine C. Coronary as well as systemic vasodilatation are mediated by opening potassium channels on vascular smooth muscle. Levosimendan is also able to reduce cardiac stunning without increasing myocardial intracellular calcium concentrations or prolonging myocardial relaxation [4],[5]. Anti-apoptotic properties in combination with a protective effect against ischemic injury in the heart mediated by activation of mitochondrial K_(ATP)_ channels have been described [6],[7]. However, current European guidelines recommend levosimendan only as ultima ratio therapy in patients suffering decompensated heart failure in a non-operative setting [8],[9]. Levosimendan has only recently been approved for perioperative usage in Germany and in consequence, its usage varies among cardiac anaesthesiologist and intensive care practitioners.

Although several animal studies suggest beneficial effects on kidney function upon administration of levosimendan [10],[11], recent data from the surgical setting are heterogeneous [12]-[14]. For instance, Bragadottir et al. reports increased cardiac output, promoted renal vasodilatation, increased renal blood flow and glomerular filtration rate (GFR) with an unchanged renal oxygen supply–demand relationship in patients receiving levosimendan [15]. In patients on a left ventricular assist device (LVAD) levosimendan application was associated with a reduced time spent on renal replacement therapy (RRT) [16]. On the contrary, levosimendan administration did not result in significant differences for creatinine, cystatin C and urine N-acetyl-b-glucosaminidase (NAG) postoperatively in patients with left ventricular ejection fraction (LVEF) below 50% undergoing coronary artery bypass grafting surgery [17]. In this context, levosimendan may be capable of protecting organ function as long as no further tissue damage has been caused during surgery. Given these suggested beneficial effects, we conducted a retrospective, observational analysis to evaluate postoperative kidney function in cardiac surgery patients with respect to timing of levosimendan administration.

## Methods

After approval of the respective ethics board (Chairperson Prof. Dr. R. Uebelhack, Study ID number: EA1-044-13; ClinGov. registration number: NCT01918618), we reviewed the charts and data derived from two electronic patient data management systems (COPRA System, Sasbachwalden, Germany and SAP, Walldorf, Germany) of all patients (≥18 years) admitted to our 28-beds ICU of the department of anaesthesiology and intensive care medicine at the Campus Charité Mitte, Berlin, between 01/01/2007 and 31/12/2011. Inclusion criteria were: a) cardiac surgery with CPB, b) no pre-existing renal dysfunction defined as creatinine plasma level > 2.0 mg/dl (SI 176.8 μmol/L) [18], and c) administration of levosimendan on the day of surgery. Levosimendan administration was triggered by a severely reduced left ventricular systolic function preoperatively (LVEF < 35%) and/or clinical signs of a low cardiac output syndrome (LCOS) intraoperatively and/or in the immediate postoperative course. Exclusion criteria were: a) incomplete medical records, b) patient age below 18. In order to evaluate postoperative kidney function, incidence of renal dysfunction defined as at least one postoperative creatinine plasma level > 1.5 times the preoperative value (based on the creatinine criterion of the KDIGO classification) and/or the need for renal replacement therapy (RRT) [19], duration of renal replacement therapy and differences in the time course of creatinine plasma levels postoperatively until day four were chosen as study endpoints.

Cardiopulmonary bypass (CPB) and anaesthesia management was performed according to our standard operating procedures [20],[21]. Normothermic CPB was established with a non-pulsatile flow of 2,5 l/min/m^2^ and an arterial pressure > 60 mmHg without additional filtration. CPB was primed with balanced electrolyte solution, hydroxyethyl starch, 1000 mg methylprednisolone and 500 mg tranexamic acid. Coagulation was offset by 400 IE•kg heparin aiming at an Activated Clotting Time (ACT) > 410 seconds. Additionally, all patients received tranexamic acid. Cardioplegic arrest was induced and maintained by intermittent administration of ante grade warm potassium enriched blood [22].

Perioperative goal-oriented haemodynamic support was established according to institutional standards guided by the German S3 guidelines [8]. Intraoperatively, patients with a preoperative reduced LVEF (<35%) and/or open cardiac surgery, e.g. valve reconstruction and/or replacement, were monitored with transoesophageal echocardiography (TEE, Vivid 7 or Vivid S6, GE, Fairfield, USA) and/or a pulmonary artery catheter with continuous mixed venous oximetry measurement. In case of difficult CPB separation despite hemodynamic optimization, an intraaortic balloon pump (IABP) was placed according to the team's assessment.

After chest closure, the patient was transferred intubated and mechanically ventilated to the ICU. Patients were kept sedated with propofol (1–3 mg/kg/h) and opioid bolus administration until cardiopulmonary stability was achieved (i.e. heart rate 80–100 beats/min, mean arterial pressure 65–85 mmHg, central venous pressure 8–12 mmHg (at positive end-expiratory pressure 5 cm H_2_O), pulmonary artery occlusion pressure 12–15 mmHg, cardiac index >2.5 l /min/m^2^, stroke volume index >30 ml/m^2^, mixed venous oxygen saturation >65), chest tube drainage was negligible (<100 ml/h), and the patient was judged to be extubated. If mechanical ventilation was required ≥ 12 hours, analgosedation was switched to midazolam 0.01 - 0.2 mg/kg/h combined with sufentanil 0.15 - 0.7 μg/kg/h, and weaning from mechanical ventilation was performed according to the standard operating procedures at our hospital [21],[23]. Hemodynamic optimization was continuously accomplished according to the German S3 guidelines [8]. The decision for RRT was taken in consultation with the department of nephrology and based on the following criteria: a) oliguria < 500 ml/dl and/or anuria < 100 ml/dl, b) metabolic acidosis, c) hyperkaliaemia, and d) uraemia.

Results are expressed as means ± standard deviation (SD), median ± quartiles or percentage, respectively. Normal distribution was checked by the Kolmogorov-Smirnov test. Differences between groups were analyzed by the Mann–Whitney U-test. Non-directional hypothesis were tested two-sided, whereas directional hypothesis were tested one-sided. By categorical scaling, the relative frequency of a variable was analyzed by the χ^2^-test. Changes in variables over time were analyzed using a nonparametric analysis of longitudinal data in a two-factorial design. Logistic regression analysis was performed to detect perioperative factors influencing renal function postoperatively. P < 0.05 was considered statistically significant. All tests should be understood as constituting exploratory data analysis, such that no adjustments for multiple testing have been made. Statistics were performed using SPSS 20.0 software (IBM Corporation, Armonk, New York, USA).

## Results

In total, 9.634 patients were admitted to our ICU during the specified study period. Thereof 4.635 underwent elective or urgent cardiac surgery, namely coronary artery bypass grafting (CABG) and/or valve reconstruction/replacement (VR). After exclusion of patients with creatinine plasma levels > 2.0 mg/dl, 1.095 patients were considered for further analyses. In this cohort, 46 patients received 12.5 mg levosimendan once as a continuous infusion in a rate of 0.1 μg/kg/min on the day of surgery without an initial bolus. 61% of the patients received levosimendan intraoperative immediately after anaesthesia induction (LEVO OR) and 39% postoperative on ICU (LEVO ICU). Thereof, the mean time interval between ICU admission and start of levosimendan administration was 1.4 hours (range 0 – 9 h).

Basic patient characteristics and surgical data are presented in Table 1. There were no significant differences in functional status, i.e. NYHA classification or other comorbidities between groups. The preoperative creatinine plasma level, the preoperative estimated GFR, number of patients under preoperative diruretic therapy, and SAPS-II admission score on ICU did not differ between groups (Table 2). Despite normal creatinine plasma values, preoperative estimated GFR was mildly reduced in both groups without statistical difference.

**Table 1:** **Morphometric and demographic data and surgical procedures**

	Levo OR	Levo ICU	***P***value
	(n = 28)	(n = 18)	
Age (years)	66 ± 10	67 ± 11	0.535
Weight (kg)	82.1 ± 15.9	81.5 ± 15.0	0.928
Height (m)	1.76 ± 0.1	1.72 ± 0.1	0.167
Body mass index (kg/m^2^)	26.3 ± 3.9	27.9 ± 5.0	0.286
Sex (men/women)	22/6	13/5	0.622
Procedure			
CABG	15	5	0.114
VR	5	8	0.051
CABG + VR	8	5	0.953
NYHA III/IV	25	16	0.966
Coronary artery disease	23	13	0.426
LVEF (%)	31 ± 12	36 ± 15	0.233
COPD	9	5	0.754
Peripheral vascular disease	7	5	0.834
Atrial fabrillation	17	9	0.474
Hyperlipidaemia	8	8	0.270
Nicotine abuse	6	1	0.144
Pulmonary arterial hypertension	12	10	0.400
Arterial hypertension	13	9	0.813
Diabetes mellitus	16	12	0.518

Data are expressed as mean ± SD, numbers or percentage. CABG, coronary arterial bypass grafting; VR, valve reconstrution and/or replacement; NYHA, New York Heart Association; LVEF, left ventricular ejection fraction; COPD, chronic obstructive pulmonary disease.

**Table 2:** **Perioperative renal function parameters and SAPS-II scores**

	Levo OR	Levo ICU	***P***value
	(n = 28)	(n = 18)	
Preoperative creatinine_plasma_ (mg/dl)	1.19 ± 0.37	1.13 ± 0.26	0.753
Preoperative eGFR (ml/min)	73 ± 23	73 ± 21	0.928
Preoperative diuretic treatment (%)	21.4	38.9	0.343
SAPS-II ICU admission	47 ± 17	44 ± 16	0.566
Incidence postoperative renal dysfunction (%)	67.9	94.4^*^	0.033
Duration of RRT (hours)	79 (35-332)	272^*^ (132-703)	0.046

Postoperative renal dysfunction is defined as a creatinine plasma level > 2.0 mg/dl. Data are expressed as mean ± SD, median and quartiles or percentage. *indicate significant differences between groups. Levo OR/Levo ICU, see text for further details; OR, operating room; ICU, intensive care unit; eGFR, estimated glomerular filtration rate; RRT, renal replacement therapy.

**Figure 1:**
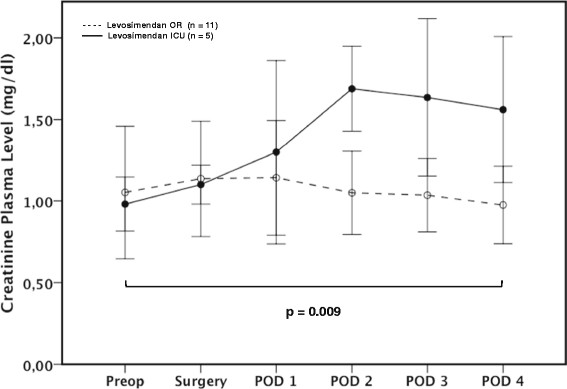
**Time course of creatinine plasma levels in patients receiving levosimendan intraoperatively or on ICU and not requiring RRT.** Postoperative creatinine plasma levels were significantly reduced when levosimendan was administered in the OR (p = 0.009). Values are expressed as means ± SD. *Preop = preoperative; Surgery = ICU admission; POD 1 – 4 = first to fourth postoperative day; LEVO OR = start of levosimendan administration after anesthesia induction in the operating room; LEVO ICU = start of levosimendan administration after admission on ICU.*

Incidence of postoperative renal dysfunction was significantly reduced in patients who received levosimendan after anaesthesia induction in contrast to those who were given levosimendan first after admission on ICU (67.9% vs. 94.4%, p = 0.033) (Table 2). In addition, early onset of treatment was accompanied by a significant reduction in creatinine plasma levels (p = 0.009) in patients not requiring RRT postoperatively (LEVO OR: n = 11; LEVO ICU: n = 5) (Figure 1) and shorter RRT duration (p = 0.046) (Table 2).

Finally, the multivariate analysis showed that early administration of levosimendan (i.e. in the operating room) had significant independent influence on postoperative kidney function (OR 0.043, 95% CI 0.002 – 0.940, p = 0.046). Also the following perioperative conditions had a significant influence on postoperative kidney function: a) peripheral vascular disease (OR 0.044, 95% CI 0.001 – 0.906, p = 0.044), b) diabetes mellitus (OR 0.013, 95% CI 0.001 – 0.257, p = 0.004).

## Discussion

This retrospective analysis suggests an association of an early – preventive – rather than a late start of levosimendan administration and postoperative kidney function in cardiac surgery patients. Levosimendan administration seemed to be triggered in order to prevent or to immediately begin treatment of LCOS in high-risk cardiac surgery patients as these patients have the highest perioperative mortality [24]. However, when a patient received levosimendan in the first course of postoperative treatment (i.e. after being admitted to the ICU), it might have been rather due to deteriorating physical conditions than preventive aspects (i.e. after anaesthesia induction). This in part might explain some of our findings. Nevertheless, patients judged to require levosimendan received the drug as "on top"-medication to the standard haemodynamic optimisation when other pharmacotherapeutical options especially on ICU (i.e. epinephrine, etc.) were exhausted.

Considering the patients of our study that received levosimendan intraoperatively, the results from our study demonstrate a positive effect on renal function. This is consistent with previous findings in patients with decompensated heart failure [25]-[27] and also inline with a recently published meta analysis in the perioperative setting [28]. The aspect of timing of levosimendan administration has been addressed by Aksun et al. [29] in a study involving 15 patients in total. They compared three groups consisting of patients that were given levosimendan at induction of anaesthesia, during weaning from CPB or postoperatively. Whereas cardiac index (CI) and pulmonary capillary wedge pressure (PCWP) improved and urine output over 24 hours was satisfactory in all groups, patients of the group that received levosimendan whilst in the ICU presented a prolonged necessity for inotropic substances and also intraaortic balloon pumps. In patients on LVAD perioperative levosimendan application was associated with a shortend RRT duration [16].

In order to detect organ protective mechanisms of levosimendan, only patients without preoperative elevated creatinine plasma levels – as a simple and frequent accessible surrogate parameter of kidney function – were included in this study. The 2.0 mg/dl limit is based on current recommendations [18]. Considering the population of this study, it would correspond to an estimated GFR of 25–35 ml/min/1.73 m^2^ and correspond to "moderately to severely decreased renal function" according to the KDIGO 2012 guidelines for staging of chronic kidney disease [30]. Interestingly, this chosen limit ruled out about ¾ of our cohort (4.635 to 1.095), indicating that most patients with a preoperatively reduced LV function were already affected by kidney dysfunction. Nevertheless, pre-existing renal impairment in our study cohort may have been masked by the already equally reduced preoperative estimated GFR in both groups. As too much irreversible organ damage might have already been manifested preoperatively, the organ protective properties of levosimendan administration in heart failure patients might be limited [31],[32].

As early as 1984, Wilson et al. demonstrated impaired skeletal muscle nutritive flow during exercise in patients with congestive heart failure although stroke volume was optimized by dobutamine infusion [33]. Consequently, improving haemodynamics is one among several aspects in the therapeutic course. Levosimendan, however, may also positively influence cell surviving beside its properties to restore haemodynamics [6],[7].

Most aforementioned studies investigated non-operative patients with decompensated heart failure. It might be possible that additional beneficial effects of levosimendan are attenuated by a systemic inflammation response syndrome (SIRS) concomitant with cardiac surgery [34],[35]. In experimental and clinical sepsis investigations, beneficial renal effects of levosimendan were only detectable if the drug improved haemodynamics [36]-[38].

This study stands out for its pragmatic clinical approach in a real life environment. As for all retrospective approaches, our analysis was limited by the variables available in routine patient care. The number of patients analyzed in this study may be regarded as relatively high given the single centre setting and the chosen eligibility criteria. Since preoperative plasma creatinine, preoperative eGFR and overall disease severity as expressed by SAPS-II admission score were statistically comparable between groups, we found it reasonable to suggest that there was no statistical bias with respect to the investigated parameters. In our analysis, we can only assume that haemodynamics were optimised as specified in the recent S3 guideline [8]. In a previous study conducted in a similar setting, there were no differences in use of norepinephrine, dobutamine and enoximone between patients receiving levosimendan or placebo [23]. Beneficial effects of norepinephrine on kidney function have been described and would thus add to the properties of levosimendan [39]. Nevertheless, additional factors like pharmalogical or mechanical support may have had a significant impact on outcome and suchlike issues need to be addressed in controlled trials. Further preoperative risk stratification using biomarkers might be another aspect of high interest for future prospective studies [40].

Up to date, the clinician's decision to administer levosimendan is mostly based on the clinical picture. Patients that received levosimendan at a later point of time may have been misjudged at first. Thus, a bias may never be completely ruled out. The fact that levosimendan is used off-label in most countries when administered in the perioperative setting also contributes to that bias. In this context it is interesting that a large double-blind, placebo-controlled, randomized, multicenter phase 3 trial is being scheduled to start in the third quarter of 2014 in North America to further investigate organ protective properties of levosimendan [41]. A multicentered phase 4 RCT trial is currently undertaken at a university hospital in Italy [42].

## Conclusions

Patients with a normal or only slightly impaired kidney function preoperatively may benefit from an early start of levosimendan administration, i.e. immediately after anaesthesia induction. Levosimendan administration in patients after admission to the ICU, mainly due to a developing or already manifest LCOS, does not seem to be beneficial to preserve kidney function. Considering the pharmacokinetics of the long acting agent, further trials are needed to evaluate assumed organ protective effects of levosimendan when given even earlier (i.e. infused one or two days preoperatively.

## Authors' contributions

All authors participated in the study management/conception/design, data collection, and interpretation of data. FB and ST were equally responsible for data collection/analysis/data interpretation, helped to prepare the study management/conception/design, and were equally responsible for drafting the manuscript. JP was responsible for the study management/conception/design and the final revision of the manuscript. KDW was responsible for statistical analysis and revising the manuscript. HG, MK, CS and MS helped in data interpretation and revised the manuscript. All authors read and approved the final manuscript for publication.

## Authors’ original submitted files for images

Below are the links to the authors’ original submitted files for images. Authors’ original file for figure 1
