# Supplementary figures and images for: Early administration of levosimendan is associated with improved kidney function after cardiac surgery – a retrospective analysis
Source: J Cardiothorac Surg. 2014 Nov 18;9:167. doi: 10.1186/s13019-014-0167-8 (PMC4240807; doi:10.1186/s13019-014-0167-8)

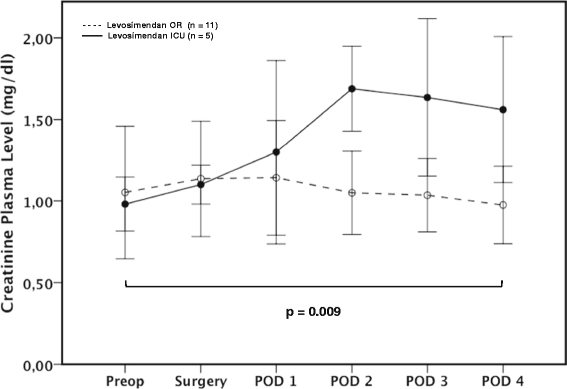

Supplement: Supplementary file 1 — Authors’ original file for figure 1 [file 13019_2014_167_MOESM1_ESM.gif]
